# Minimally invasive approach to Dunbar Syndrome

**DOI:** 10.12669/pjms.42.8.12313

**Published:** 2026-08

**Authors:** Sajida Qureshi, Mohammad Fahad Tariq Barlas, Fahad Memon, Sadia Jamal

**Affiliations:** 1 Sajida Qureshi, FCPS, FRCS Professor of Surgery, Dow Medical College, Dow University of Health Sciences, Karachi, Pakistan; 2 Mohammad Fahad Tariq Barlas, Assistant Professor, Department of Vascular Surgery, SMBBIT, Karachi, Pakistan; 3 Fahad Memon, Resident General Surgery, Civil Hospital Karachi (CHK), Karachi, Pakistan; 4 Sadia Jamal, MBA, BBA. University of Karachi, Dow University of Health Sciences, Karachi, Pakistan

**Keywords:** Median arcuate ligament, Minimally Invasive Surgery, Dunbar syndrome, Median arcuate ligament syndrome

## Abstract

Median arcuate ligament syndrome (MALS) is a rare vascular condition, more commonly observed in females and typically considered a diagnosis of exclusion. Its clinical presentation often mimics chronic mesenteric ischemia, making diagnosis challenging. We report an interestingly rare case of a young male presenting with chronic postprandial abdominal pain, nausea, and significant weight loss. Following an extensive diagnostic workup and exclusion of other potential etiologies, a diagnosis of MALS was established. The patient underwent successful laparoscopic decompression of the celiac artery with division of the median arcuate ligament, resulting in complete symptom resolution.

## INTRODUCTION

Median arcuate ligament syndrome is also known as celiac artery compression syndrome or Dunbar syndrome, an extremely rare cause of chronic gut ischemia with an incidence of 2 out of 100,000 patients. Compression of the celiac artery by the median arcuate ligament is responsible for the symptoms. Predominantly observed in women between the ages of 40 and 60, the course of this condition often includes weight loss and the development of severe malnutrition.^1^ The celiac axis originates from the abdominal aorta, from T11 to L1, although its initiation point can vary.^2^ The diaphragmatic crura, emerging from L1–L4, converge at the anterior and superior aspect of the celiac artery. The Median arcuate ligament is a fibrous band that connects the diaphragmatic crura encircling the aortic hiatus at the anterior side. In cases where the celiac artery commences at a higher point or the diaphragmatic crura insert at a lower position, it may result in Median Arcuate Ligament Syndrome (MALS). Additionally, there are occurrences where the median arcuate ligament crosses the lower abdominal aorta, inducing compression.^3^

## CASE PRESENTATION

A 28-years-old male laborer, resident of Karachi, presented to our department with chronic, recurrent, postprandial upper central abdominal pain for eight years and undocumented weight loss. The pain was gradual in onset, dull, and in the upper central abdominal region. It was postprandial, moderate to severe, and was occasionally relieved by painkillers. It was non-radiating and non-shifting and associated with nausea on and off. He also had constipation on and off. The patient had no other comorbidities and had an Eastern Cooperative Oncology Group (ECOG) performance status of 0. The rest of the history was unremarkable. In the past years, all the workups done for evaluation of his symptoms, including baseline tests and ultrasounds, were unremarkable. Later a CT scan of the abdomen and pelvis was done to aid in diagnosis.

On general physical examination, the patient was of average height and lean build, with vital signs well within range and with no remarkable findings except for weight loss of 12 kilograms since the onset of symptoms. On abdominal examination, a scaphoid abdomen was noted with abdominothoracic respiration, no visible scar, veins, pulsation, or mass. The abdomen was soft, nontender to palpation, and no visceromegaly was noted. Gut sounds were audible with no bruit appreciable. Examination of the chest was unremarkable, with bilaterally equal air entry and normal vesicular breathing. On CT scan of abdomen and pelvis, focal narrowing of the celiac artery near its origins was noted, giving a hooked appearance on sagittal images. This finding was likely due to indentation of the superior surface of the celiac trunk by the median arcuate ligament, raising the suspicion of median arcuate ligament syndrome. The rest of the CT scan showed no significant abnormality. With the working diagnosis of median arcuate ligament syndrome, the patient was referred to CT angiogram.

**Fig.1:**
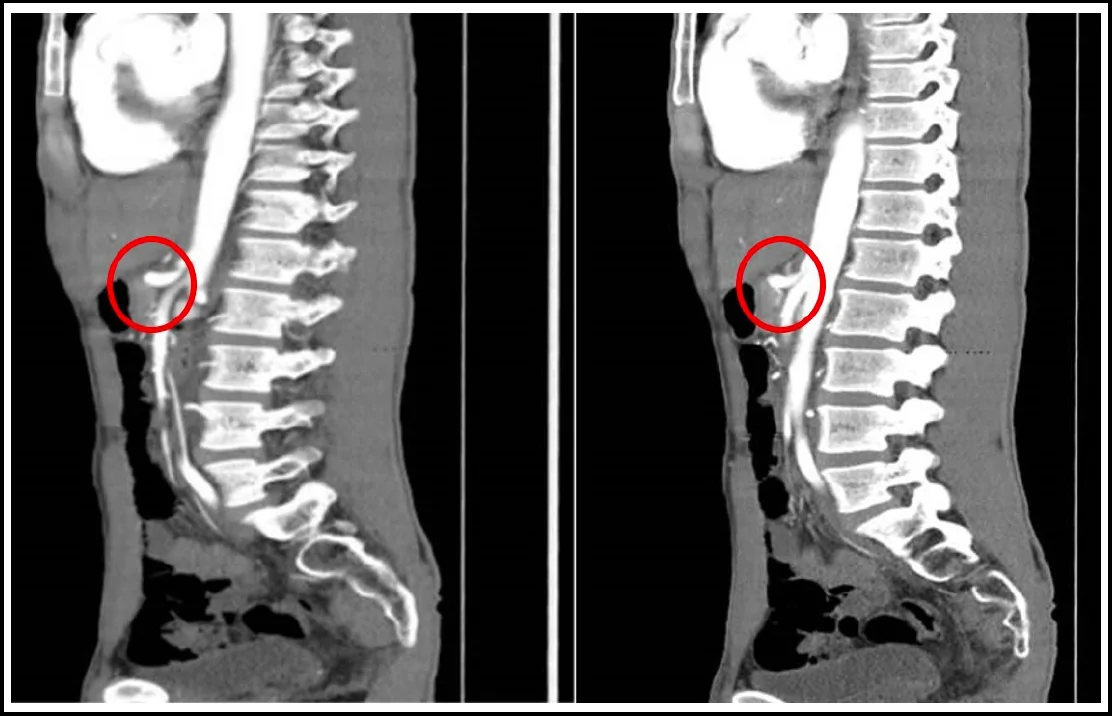
Computed Tomography Scan.

CT angiogram of the abdominal aorta showed focal stenosis of the celiac trunk on its superior surface with a hooked appearance, likely because it’s indented by a low lying median arcuate ligament. The rest of the CT angiogram was unremarkable (Fig.1). Based on these findings, the patient was diagnosed with median arcuate ligament syndrome, also called celiac artery compression syndrome and Dunbar syndrome. The patient underwent laparoscopic decompression of the celiac artery by division of the median arcuate ligament, and recovered well postoperatively, and was discharged the next day. There was a complete resolution of symptoms in the first week of follow up, no postprandial pain in the second week of follow up. A month later there was still no active complaint and three months later, the patient remained symptom free with a weight gain of 2.5 kilograms.

### Procedure

**Fig.2:**
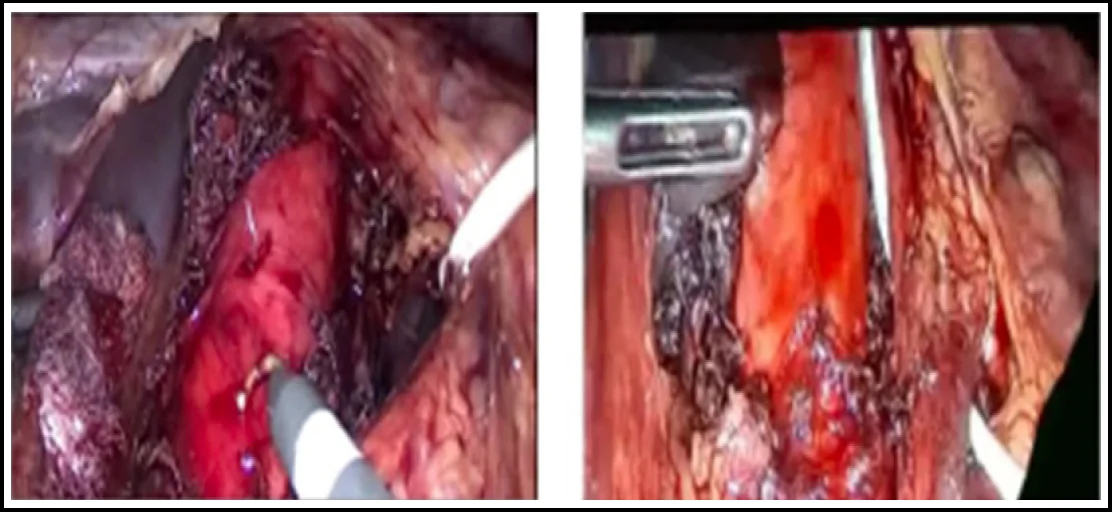
Intraoperative images of the procedure.

Patient was placed in modified lithotomy French position with surgeon standing between the legs. Closed technique of creating pneumo-peritoneum at the Palmers point with Veress needle was employed. One camera port and four working/assistant ports were inserted. A 12-mm umbilical camera port, one 10-mm port in the left upper abdomen, and two 5-mm ports in the right upper abdomen were inserted. Nathanson retractor was placed in the epigastric region to retract the left liver lobe, Laparoscopy was performed with a 30° laparoscope, a Ligasure was used to open the gastro-hepatic ligament and dissect between the right diaphragmatic crus and the gastric ligament. The left gastric vein and artery were identified and taped. The median arcuate ligament (MAL) and nervous plexus around the celiac artery were identified, and the thickened diaphragmatic crura were exposed. The MAL and the nervous plexus were then dissected, and the dissection line was continued to the front of the abdominal aorta. The diaphragmatic fibers overlying the anterior surface of the abdominal aorta were divided, and the dissection was carried caudally along the anterior aspect of the aorta until the origin of the celiac truck was identified. The median arcute ligament fibers and celiac plexus were then divided, and the proximal celiac artery was circumferentially skeletonized to achieve complete decompression (Fig.2). Overall, the intraoperative time was about 150 minutes with minimal blood loss of 200 ml.

## DISCUSSION

Median arcuate ligament syndrome demonstrates significant anatomical variability, and since approximately 85% with celiac artery compression remain asymptomatic, anatomical compression alone is not believed to drive symptom development.^4^-^6^ The hemodynamic factors such as significant celiac stenosis is likely contributing to clinical presentation as observed in our patient.

The Pathophysiology is poorly understood and has been proposed to involve both ischemic and neuropathic mechanisms. Some literature suggests it might be both, though the evidence is conflicted.^2^ Usually, the clinical features are a triad of abdominal pain (postprandial), weight loss and variable degrees of nausea and vomiting. Weight loss is often unintentional. Physical examination may be normal; however weight loss might be apparent, and there may be epigastric tenderness and epigastric bruit. The epigastric bruit is louder on expiration but not always present, seen in about a third of patients with MALS. Also, it’s not specific to MALS.^7^

Our patient had the clinical features of chronic, recurrent postprandial abdominal pain, nausea and weight loss, but there was no tenderness or bruit on examination. Thus, there was very little hint on history and clinical examination to suggest the cause of his symptoms might be MALS. The only clue was the inconclusive baseline workups done previously and it has been described in literature that MALS is a diagnosis of exclusion since it is so rare.

Radiologic tools such as CT angiography are crucial in diagnosing MALS, which shows a characteristic hooked appearance. This hooked appearance was noted in our patient, with no other remarkable findings and we confirmed the diagnosis of MALS based on the CTA findings.

The first line treatment of MALS is by surgery, either open or by minimally invasive approaches. Laparoscopic and open division of MALS had similar outcomes (minimal morbidity and mortality) although with laparoscopic approach, patients resumed feeding faster and hospital stay was reduced.^8^ Appropriate patient selection is of the utmost importance while treating patients with median arcuate ligament syndrome. More success is associated with patients who are between 40 to 60 years, have post prandial abdominal pain pattern and have a weight loss of more than twenty pounds. Less success is associated with patients who have an atypical pattern of pain, age above 60 years, weight loss less than twenty pounds and a history of psychiatric disorder or of alcohol abuse. Thus, keeping our case in mind, we proceeded with surgery.

We performed laparoscopic decompression of the celiac artery with median arcuate ligament release, and as literature has proved,^2^ our patient too, had a good postoperative recovery and was discharged from the hospital on the very next day of surgery with no postoperative complications. On follow up, the patient had complete resolution of symptoms, with no recurrence or complains even on follow up at three months, with an added weight gain of 2.5 kgs, which was good, seeing that his weight loss was unintentional to begin with.

There is a need to investigate how celiac artery compression syndrome is symptomatic in certain patients and not in others. Perhaps the incidence of median arcuate ligament is so less because it may remain undiagnosed or misdiagnosed, perhaps throughout a patient’s lifetime. We thus may never know the true incidence of median arcuate ligament syndrome in Pakistan. Moreover, the exact cause is still obscure though many theories exist.

## CONCLUSION

A rare disorder, median arcuate ligament syndrome, is often misdiagnosed or remains undiagnosed since the symptoms are nonspecific and the tests that diagnose it are not routinely done. A CT angiogram can diagnose the syndrome based on specific findings. No known medical treatment exists, and given the proposed causes and keeping in mind the clinical anatomy, surgical release is the treatment of choice. It must be done with great care as there is a risk of vascular injury. The outcomes after surgery are favorable, with complete resolution of symptoms as observed in our case patient.

### Author’s Contributions:

**SQ:** Concept, design, surgery, drafting, critical appraisal, approval of the case report and is responsible and accountable for the accuracy and integrity of the work.

**MFTB:** Data collection, Review, Follow-up.

**FM:** Writing, Patient Follow-up.

**SJ:** Literature search, Editing, Writing.
